# The Multifaceted Roles of Biomarkers in Metabolic Disorders: From Diagnosis and Monitoring to Prediction, Management, and Treatment

**DOI:** 10.3390/metabo16100776

**Published:** 2026-10-13

**Authors:** Prince Dadson, Miikka-Juhani Honka

**Affiliations:** Turku PET Centre, Turku University Hospital, University of Turku, 20520 Turku, Finland

## 1. Introduction

The rising prevalence of obesity, type 2 diabetes, and cardiovascular disease has made the identification of reliable biomarkers a central priority in metabolic research [1,2]. Functional biomarkers, including metabolites, microRNAs, and proteins, can detect pathological dysfunction before overt disease becomes clinically apparent [3,4]. Their systematic study can also help provide detailed information on the biochemical pathways through which drugs, nutrients, and environmental exposures alter metabolic homeostasis [5,6]. Advances in analytical chemistry, particularly liquid chromatography and mass spectrometry-based metabolomics, now enable tissue-specific and disease-specific metabolic signatures to be characterized with a level of precision that was not achievable a decade ago [7,8].

Biomarkers capable of predicting treatment response, rather than simply confirming the presence of disease, remain limited for many conditions. The metabolic consequences of comorbidities, such as autoimmune disease accompanying endocrine dysfunction or obesity accompanying inherited metabolic disease, are often poorly captured by biomarker panels developed in more homogeneous cohorts. The translation of multi-omic biomarker data into personalized management algorithms also remains at an early stage across most metabolic disorders. This Special Issue, “The Multifaceted Roles of Biomarkers in Metabolic Disorders: From Diagnosis and Monitoring to Prediction, Management, and Treatment,” brings together six contributions addressing these challenges from complementary perspectives, including clinical pharmacology, lipidomics, rare inherited diseases, hepatic mitochondrial biochemistry, and the metabolic and skeletal complications of chronic kidney disease (CKD).

## 2. Special Issue Contributions

This Special Issue comprises six articles on biomarkers in chronic kidney disease, pregnancy, endocrine disorders, inherited metabolic diseases, and mitochondrial metabolism. Although the conditions studied differ, the articles address a common question: how biomarkers in metabolic medicine can be used beyond diagnosis, for risk stratification, treatment monitoring, and investigation of disease mechanisms.

Hyperprolactinemia and autoimmune thyroiditis are associated with increased cardiometabolic risk, and these conditions frequently co-occur in premenopausal women. A dopamine agonist, cabergoline, can attenuate cardiometabolic risk factors in individuals with hyperprolactinemia, but this beneficial effect is blunted among young women with autoimmune thyroiditis. Autoimmune thyroiditis is also common after menopause; however, hyperprolactinemia is difficult to diagnose because classical symptoms can be absent. Krysiak and colleagues (Contribution 1) assessed whether autoimmune thyroiditis modifies the cardiometabolic response to cabergoline in postmenopausal women with hyperprolactinemia. Reductions in uric acid, high-sensitivity C-reactive protein, fibrinogen, and homocysteine were statistically significant only among women without thyroid autoimmunity. This interpretation is independent of thyroid-stimulating hormone status, although a causal role for thyroid autoimmunity-associated inflammation was not established.

Pregnancy causes significant metabolic changes in the mother. However, the changes in plasma metabolome have not been comprehensively characterized over the progression of pregnancy. Traila and colleagues (Contribution 2) used UHPLC-QTOF-ESI^+^-MS lipidomics to characterize plasma metabolites in pregnant women sampled longitudinally across three gestational windows and in postpartum women. Among the identified metabolites, estrogen derivatives, long-chain fatty acids, and ceramides discriminated pregnancy from the postpartum state. A similar group of lipid species, together with dihydrocorticosterone and taurine, distinguished early from late pregnancy. These findings identified a broader group of lipid metabolites associated with gestational stage.

Gaucher disease type 1 is a rare genetic disorder caused by deficient activity of β-glucocerebrosidase, resulting in the accumulation of glucosylsphingosine (Lyso-Gb1) in multiple organs such as spleen and liver. This disease has been conventionally treated using intravenous enzyme replacement therapy; however, this is hampered by challenging dosing, costs, and logistics. Eliglustat is a relatively new oral drug for Gaucher disease that inhibits glucosylceramide synthase, thereby reducing the synthesis of glucosylceramide and downstream sphingolipid metabolites, including Lyso-Gb1. It addresses most of the challenges of enzyme replacement therapy, but the possible effects of obesity and splenectomy on the treatment’s efficacy remain incompletely understood. Maines and colleagues (Contribution 3) reported the clinical course of an obese, splenectomized patient with Gaucher disease type 1 who developed a progressive increase in plasma and dried blood spot Lyso-Gb1 after switching from long-term enzyme replacement therapy to eliglustat, despite documented treatment adherence. The biochemical changes were accompanied by worsening fatigue and a marked increase in plasma chitotriosidase activity. Both clinical and biochemical abnormalities improved within months of reinstating enzyme replacement therapy.

Hepatic mitochondria have a key role in systemic nutrient balance, nitrogen detoxification, and hepatocyte bioenergetics. Genetic defects affecting hepatic mitochondrial pathways may result in inborn errors of metabolism. Erdal and colleagues (Contribution 4) reviewed hepatic mitochondrial amino acid catabolism, including the glycine cleavage system, glutamate dehydrogenase, proline and lysine degradation, branched-chain amino acid oxidation, and urea cycle metabolism, together with the associated inborn errors of metabolism and their molecular genetic basis. Their review also addresses mitochondrial amino acid transporters involved in hyperornithinemia–hyperammonemia–homocitrullinuria syndrome and citrin deficiency, while also summarizing disease-causing variants, biochemical markers, and emerging therapeutic approaches, including gene therapy and genome editing.

Osteoporosis is a common but underappreciated comorbidity among patients with CKD. Gembillo and colleagues (Contribution 5) reviewed the epidemiology, pathophysiology, diagnosis, and management of fracture risk in CKD. Their review discussed the underuse of bone mineral density assessment, trabecular bone score, and biochemical markers in CKD and considered treatment strategies incorporating antiresorptive and anabolic agents within updated KDIGO recommendations.

Oxidative stress can promote the formation of advanced glycation end products (AGEs), which contribute to tissue inflammation, fibrosis, and endothelial dysfunction and are implicated in cardiovascular and renal disease. In healthy humans, AGEs are catabolized and excreted by the kidneys; however, in CKD, these functions are compromised. Glycated albumin is the most common circulating early glycation product, and as such can be a precursor for AGEs. Vettoretti and colleagues (Contribution 6) investigated glycated albumin as a biomarker in patients with CKD. Glycated albumin was associated with glycemic status and renal function but not with advanced glycation end products (AGEs), AGE receptor signalling, or inflammatory markers. These findings suggest that glycated albumin primarily reflects glycemic status rather than AGE-related oxidative and inflammatory activity in CKD, which is relevant to its interpretation in patients with impaired renal function.

## 3. Challenges and Future Directions

Collectively, the six papers in this Special Issue demonstrate the range of contemporary biomarker research in metabolic disorders. From glycemic monitoring in CKD and lipidomic profiling in pregnancy to assessment of therapeutic response, mitochondrial metabolism, skeletal complications, and rare disease management, these contributions illustrate how biomarkers are evolving from isolated measurements into integrative tools that connect pathophysiology with patient care.

Autoimmune thyroiditis was associated with an attenuated cardiometabolic response to cabergoline, while obesity and prior splenectomy were identified as potential factors associated with an altered Lyso-Gb1 response to eliglustat. In chronic kidney disease, glycated albumin was not associated with measures of AGE accumulation or inflammatory activity, indicating that the same measurement may have a different biological interpretation in this population. Longitudinal and stage-specific sampling, as applied to gestational lipidomics and CKD stages G3a to G5D, further captured biomarker trajectories that cross-sectional studies cannot resolve.

The two reviews (Contributions 4 and 5) demonstrate the value of narrative synthesis as a complement to primary biomarker research, particularly when biochemical and imaging markers are dispersed across small case series and single-centre cohorts. This is particularly relevant to inherited disorders of mitochondrial amino acid metabolism and CKD-associated bone fragility, where the available evidence remains fragmented.

Regarding areas for further research, adequately powered multicentre studies are needed to clarify the relevance of body composition, splenectomy status, and pharmacogenomic variation to individualized dosing and treatment response in Gaucher disease. Prospective studies should also determine whether Lyso-Gb1 can identify early loss of treatment efficacy rather than serving primarily as a marker of an established change in disease activity, as suggested by the case reported here. The gestational lipidomic signatures require validation in independent cohorts, including women at risk of preeclampsia, gestational diabetes, and fetal growth restriction, before clinical application can be considered. The apparent interaction between thyroid autoimmunity and response to dopamine agonists also requires confirmation in randomized trials. In CKD, prospective studies should establish the prognostic value of glycated albumin for cardiovascular outcomes independent of HbA1c and determine whether incorporating bone mineral density and trabecular bone score into CKD-MBD care pathways reduces fracture incidence rather than simply increasing case detection. Contribution 4 identifies glutamate dehydrogenase, glutaminase 2, and carbamoyl phosphate synthetase 1 as enzymes implicated in both inherited mitochondrial disease and tumour metabolism. Their potential as therapeutic targets in either setting remains to be fully established.

Taken together, these contributions demonstrate how functional assessment of biomarkers can improve diagnosis, monitoring, and prediction of treatment response across endocrine, reproductive, and renal metabolic disorders. We thank the authors for their contributions and the reviewers for their careful assessment of the submitted manuscripts.
